# Molecular characterization of the insecticidal activity of double-stranded RNA targeting the smooth septate junction of western corn rootworm (*Diabrotica virgifera virgifera*)

**DOI:** 10.1371/journal.pone.0210491

**Published:** 2019-01-10

**Authors:** Xu Hu, Joseph P. Steimel, Deirdre M. Kapka-Kitzman, Courtney Davis-Vogel, Nina M. Richtman, John P. Mathis, Mark E. Nelson, Albert L. Lu, Gusui Wu

**Affiliations:** 1 DuPont Pioneer, Johnston, Iowa, United States of America; 2 DuPont Pioneer, Hayward, California, United States of America; Chinese Academy of Agricultural Sciences Institute of Plant Protection, CHINA

## Abstract

The western corn rootworm (WCR, *Diabrotica virgifera virgifera*) gene, *dvssj1*, is a putative homolog of the *Drosophila melanogaster* gene, snakeskin (*ssk*). This gene encodes a membrane protein associated with the smooth septate junction (SSJ) which is required for the proper barrier function of the epithelial lining of insect intestines. Disruption of DVSSJ integrity by RNAi technique has been shown previously to be an effective approach for corn rootworm control, by apparent suppression of production of DVSSJ1 protein leading to growth inhibition and mortality. To understand the mechanism that leads to the death of WCR larvae by *dvssj1* double-stranded RNA, we examined the molecular characteristics associated with SSJ functions during larval development. *Dvssj1* dsRNA diet feeding results in dose-dependent suppression of mRNA and protein; this impairs SSJ formation and barrier function of the midgut and results in larval mortality. These findings suggest that the malfunctioning of the SSJ complex in midgut triggered by *dvssj1* silencing is the principal cause of WCR death. This study also illustrates that *dvssj1* is a midgut-specific gene in WCR and its functions are consistent with biological functions described for *ssk*.

## Introduction

RNA interference (RNAi) pathways are common among many eukaryotes including insects [1], and transgenic crops utilizing RNAi represent a promising new tool for insect pest management [2]. The western corn rootworm (WCR), *Diabrotica virgifera virgifera* (Coleoptera: Chrysomelidae), is one of the most economically important pests of maize in the United States, and Europe where it is an invasive species [3, 4]. Currently, WCR damage is managed by combinations of crop rotation, broad-spectrum soil insecticides [5], and transgenic crops expressing crystalline (Cry) proteins from *Bacillus thuringiensis* (Bt) [4]. Insect resistance to existing transgenic traits threatens the durability of Bt crops[6], which highlights the importance of developing new technologies for protection from WCR [7, 8].

The general mechanism through which RNAi works has been well described [9], as has its potential for a new transgenic approach to rootworm control [2, 10]. Many genes have been reported to be effective insecticidal targets in WCR following the provision of double-stranded RNA (dsRNA) in diet bioassay [11–13]. Effective control of WCR in transgenic maize plants has been achieved with RNAi targeting the *α-tubulin* gene [11], the *V-ATPase* subunit A [11] and C [14] genes, an intracellular protein trafficking pathway gene, *snf7* [11, 15, 16], and a midgut expressed gene, *dvssj1*[17]. Once dsRNA is ingested by a susceptible insect and taken up by the midgut epithelial cells, dicer RNase III type enzymes bind and digest cytoplasmic dsRNA into small RNAs (siRNAs), that then associate with an RNA-induced silencing complex (RISC) and lead to specific suppression of the target mRNA [18]. While it has been reported that WCR larvae mortality can be caused by *Dvsnf7* [15] and *Dv v-ATPase C* [14] dsRNAs, this RNAi response was observed only with dsRNAs greater than 60 bp in length. A lack of uptake of short *Dvsnf7* dsRNA (<60 bp) or 21 bp siRNA was observed in larval midgut cells [15]. Previously, we reported that both dietary delivery of dsRNA and transgenic plants expressing dsRNA targeting *dvssj1* result in mRNA suppression leading to growth inhibition and mortality of WCR by apparent suppression of smooth septate junction (SSJ) protein located within the intestinal lining [17].

The epithelia of most invertebrate species possess specialized cell-cell junctions, known as septate junctions (SJ) [19, 20], that typically form circumferential belts around the apicolateral regions of epithelial cells and form the paracellular barrier between adjacent cells [20]. Arthropods have two types of SJs: pleated SJs (PSJs) and smooth SJs (SSJs), found in ectodermally and endodermally derived epithelia, respectively [21]. SSJs appear to regulate the paracellular pathway of the intestine and renal system in arthropods [22]. The molecular composition of SSJs is different from that of PSJs[21]. More than 20 PSJ-related proteins have been identified and characterized in *Drosophila melanogaster* [21]. In contrast, only three SSJ-specific proteins encoded by *D*. *melanogaster* genes snakeskin (*ssk*), *mesh* and tetraspanins (*tsp2A*) have been reported [23–25]. SSK, MESH, and TSP2A form a complex that are mutually interdependent for their correct SSJ localization [24, 25]. Several PSJ components, including Discs large (Dlg), Lethal giant larvae (Lgl), Coracle (Cora) and Fasciclin III (FasIII), have been confirmed to localize to SSJs. In *ssk*-and *mesh*-deficient midguts, Lgl, Cora, and FasIII are mislocalized but Dlg is not [24]. The functions of these PSJ proteins in SSJs remain uncertain since Dlg, Lgl, Cora, and FasIII are not required for the SSJ localization of TSP2A, MESH, and SSK, and are dispensable for SSJ formation [21]. Genetic studies in *D*. *melanogaster* have shown that normally impermeant fluorescent-labeled dextrans (10 kDa) are able to access the paracellular route in mutant flies that are defective for smooth septate formation[21]. The *ssk*-RNAi and *ssk*-deletion mutants are lethal at the late stage 17 of *D*. *melanogaster* embryo. *Ssk*, *mesh*, and *tsp2A* are essential to *D*. *melanogaster* development, SSJ formation, and midgut paracellular barrier function [23–25].

The goal of the current study is to understand the mechanism of dsRNA insecticidal activity when targeting the SSJ of WCR by analyzing: (1) silencing effects on molecular expression as it relates to larval growth and survival; and (2) *dvssj1* function by replacing *ssk* with *dvssj1* in *D*. *melanogaster* utilizing CRISPR/Cas9 gene modification. Our results show that compromised *dvssj1*/*ssk* expression caused by either RNAi- or CRISPR/Cas9-mediated systems is associated with defects in SSJ structure, changes in the localization of certain SSJ proteins, and impaired barrier function of the midgut in both WCR and *D*. *melanogaster* strains which culminate in impaired larval development and survival.

## Method and materials

### Double-stranded RNA production by *in vitro* transcription

The gene-specific primers (S1 Table) contained T7 RNA polymerase sites at the 5’ end of each primer were used to generate PCR product that served as the template for dsRNA synthesis by *in vitro* transcription (IVT) using a MEGAscript kit (Life Technologies, Carlsbad, CA). For Cy3-labelling of dsRNA, 25% of CTP and UTP were replaced with Cy3-CTP and Cy3-UTP (GE Healthcare, UK). DsRNAs were purified by Megaclear kit (Life Technologies, Carlsbad, CA) and examined by 48-well E-gel electrophoresis (Life Technologies) to ensure dsRNA integrity and quantified using Phoretix 1D (Cleave Scientific) or a NanoDrop 8000 Spectrophotometer (ThermoFisher Scientific,).

### Target expression during WCR different life stage

To assess levels of *dvssj1* mRNA and protein expression at different life stages, WCR rearing, total RNA extraction, and complementary DNA (cDNA) synthesis were prepared as in a previous study [26]. WCR were reared on artificial diet, collected at different life stages and/or kept in 10% neutral buffered formalin (4% formaldehyde) for 48 to 72 hours and processed for paraffin embedding.

### Quantitative reverse transcription PCR (qRT-PCR)

Gene expression was analyzed using two-step real-time quantitative RT-PCR. The assay was run, with 3 replicates per sample, using a singleplex set up with Bioline Sensifast Probe Lo Rox kit (Taunton, MA) and analyzed using the 2-^ΔΔCt^ method based on relative expression of the *dvssj1* gene and a reference gene *dvrps10* (S1 Table). Data from qRT-PCR assays were analyzed using JMP (Version *12*. SAS Institute Inc., Cary, NC) and statistical differences were detected using one-way analysis of variance (ANOVA) followed by Tukey’s multiple comparison tests; *P*<0.05 was considered statistically significant.

### *In situ* hybridization and immunohistochemistry analyses

Paraffin sections were cut 5 μm thick, collected on Superfrost Plus Gold slides (Fisher Scientific), air-dried overnight, and baked for 1 hour at 60°C. Sections were processed for RNA *in situ* hybridization (ISH) with the RNAScope Detection Kit (Chromogenic) according to the manufacturer’s standard protocol (Advanced Cell Diagnostics, Hayward, CA) or as in a previous study [26]. Slide images were acquired using a Leica Aperio CS2 digital scanner and captured at 40x magnification with a resolution of 0.25 μm per pixel. For immunohistochemistry (IHC), sections were incubated with primary antibodies (1:500 dilution) at 4°C overnight as described in S1 Method. Following five washes, they were incubated with secondary antibodies (goat anti-mouse antibody Alexa Fluor 488; 1:500 dilution) for overnight. After five additional washes, they were coverslipped and imaged with a confocal laser scanning microscope (TCP SP2; Leica) or EVOS FL Auto Imaging System (ThermoFisher) at room temperature.

### Correlation between molecular expression and larval dose-response bioassay

#### Larval bioassays

Larval bioassays were conducted in three independent replicates, with each replicate consisting of nine treatments: two negative controls (water & *Escherichia coli* GUS dsRNA) and seven *dvssj1* dsRNA doses. Overall diet preparation method followed a modified version of the manufacturer’s recommendations for *Diabrotica* artificial diet (Frontier Scientific, Inc., Newark, DE) [27]. Two 96-well diet plates per treatment were prepared as described previously [17], with seven dosing solutions containing purified *dvssj1* dsRNA at concentrations of 1.7 to 1.7×10^−6^ ng/μl. Control diet containing GUS dsRNA was prepared at the highest dose of 1.7 ng/μl. Newly hatched WCR were acclimated to neutral diet for 24 hours prior to transfer to treatment plates at infest rates of either 1 per well or 1–3 per well for all treatments. Infested plates were sealed in bags containing moist paper towels and placed in an incubator (Percival Scientific, Inc., Perry, IA) set at 27.5°C and 0:24 light to dark hours. Plates infested with 1–3 insects per well were collected after 48 hours, assessed for development based on head capsule size [28] and mortality, and live insects were used for analysis of *dvssj1* transcript and protein levels. Plates infested with 1 insect per well were assessed at the end of seven days for growth inhibition as previously described [17], along with development and mortality.

#### RNA isolation and qRT-PCR

Insects were collected from larval bioassays for *dvssj1* expression analysis at 48 hours post-dsRNA-exposure. Three samples per treatment per bioassay replicate were collected at a rate of 15 live whole insects per sample, homogenized in Buffer RLT from the RNeasy Mini Kit (Qiagen N.V., Hilden, Germany), and stored frozen at -80°C. Total RNA isolation was guided by the kit-provided protocol with an on-column DNase treatment (RNase-free DNase kit, Qiagen N.V.). Total RNA was quantified and cDNA was prepared on the day of RNA isolation using the SensiFAST cDNA Synthesis Kit (Bioline, London, England). Reaction conditions were as described in kit instructions, and reverse transcription (RT) was conducted in a C1000 Touch instrument (Bio-Rad Laboratories, Inc., Hercules, CA). Resulting cDNA was analyzed by a MIQE-compliant triplex assay between *dvssj1* and two genes (*α-tubulin* and elongation factor 1a, *EF1α*) previously validated as suitable RT-qPCR references in WCR [29–31]. The SensiFAST Probe Lo-ROX Kit (Bioline) was used to amplify cDNA on 384-well PCR microplates (Corning, Inc., Corning, NY). In addition to insect samples, each plate contained three standard curves of synthetic DNA fragments (one per assay target) and no amplification controls. All standards, controls, and samples were loaded in triplicate. Quantitative PCR was performed using the Life Technologies Viia 7 Real-Time PCR System (ThermoFisher Scientific, Inc.).

#### Gene expression analysis

Quantitative PCR data were generated using the QuantStudio Real-Time PCR Software (v. 1.1) (ThermoFisher Scientific, Inc.) with a standard curve experimental design. Expression of *dvssj1* in each treatment group was determined by first interpolating concentration values from the appropriate standard curve, then calculating a ratio between the value obtained for the target gene relative to each reference gene within each sample. The geometric mean of the ratios within a sample was determined and the natural log of each resulting sample value was calculated. All log-transformed values within a treatment group were averaged and the final value per treatment was determined by back-transforming the difference between the treatment and water control group and multiplying by 100 to express final values as a percentage of the water control group (S5 and S7 Figs). These calculations were performed within each of the three bioassay replicates and final values from all three replicates were plotted using Prism 7 (ver. 7.02, GraphPad Software, Inc., La Jolla, CA).

#### Western blot analyses

Extracted guts from 15 larvae were combined and homogenized in 15μl of ice-cold buffer (50 mM Na_2_HPO4-NaH_2_PO_4_, 50 mM NaCl, 5 mM EGTA, 5 mM EDTA, pH 7.5) that contained Complete protease inhibitors at 2.2X standard concentration (Roche) and 1mM PMSF using a hand-held motorized homogenization Pellet Pestle (Thermo Cat # 12-141-361 and Kimble 749520–0000). Homogenate (10 μl) was mixed with sample buffer 26 μl for the carcass of 2X NuPAGE LDS Sample Buffer and Reducing Agent (Life Technologies, Carlsbad, CA) and heated to 100°C for 10 min. Prepared samples were separated on 4–12% Bis-Tris polyacrylamide gels (NuPAGE, Life Technologies) using MES running buffer (NuPage, Life Technologies). Proteins were then transferred to nitrocellulose membrane using an iBlot2 system (Life Technologies, Carlsbad, CA). After transfer, membranes were blocked overnight in 5% non-fat milk in PBS, 0.1% Tween-20, and 5% glycerol. Membranes were then incubated with anti-DVSSJ1 mouse antibody (Genescript, USA) at 1: 2,500 in 1% non-fat milk in PBS with 0.1% Tween-20 for at least 90 minutes, followed by 1-hour incubation in HRP-conjugated goat anti-mouse (GAM) secondary Ab at 1: 12,500 (Bio-Rad, Hercules, CA). Western blots were developed for 5 minutes using SuperSignal West Dura Extended Duration Substrate (Life Technologies, Carlsbad, CA). Band densities from chemiluminescence were measured after digital imaging with a LAS-4000 system (GE Healthcare, Pittsburg, PA) using the TotalLab software suite (Nonlinear Dynamics, Durham, NC). Protein concentrations for midgut homogenates were determined using Pierce Coomassie Plus Assay with BSA standards (ThermoFisher Scientific, Waltham, MA). Western blot band volumes for each treatment were adjusted for total protein loaded per sample and then normalized to the values determined for water treated control samples. The data were plotted and analyzed using Prism 7 (ver. 7.02, GraphPad Software, Inc., La Jolla, CA). The concentration-response data for transcript and protein expression were fit using a standard logistic equation (y = A1 + (A2-A1)/(1 + 10^((LOGx0-x)*p).

### siRNA profile analysis in WCR larvae exposed to *dvssj1* dsRNA in diet

WCR neonates were transferred to artificial WCR diet containing 210-bp *dvssj1* dsRNA at 180 ng/μl in the diet for 48 hours and collected for total RNA isolation. Total RNA was prepared using the mirVana RNA isolation kit (Life Technologies), and 1μg of RNA used to generate small RNA libraries using the TruSeq small RNA kit (Illumina). RNA 3′ and 5′ adapters were ligated in consecutive reactions with T4 RNA ligase. Ligated RNA fragments were primed with an adapter-specific RT primer and reverse transcribed with Superscript II reverse transcriptase (Life Technologies) followed by eleven cycles of amplification with adapter specific primers. Resulting cDNA libraries were separated on a 6% TBE gel and library fragments with inserts of 15–50 bp excised. Recovered cDNA libraries were validated by QC on an Agilent Bioanalyzer HiSens DNA chip (Agilent Technologies Inc.) and were sequenced for 50 cycles on the Illumina GAIIx according to Illumina protocols with one sample per lane. Trimmed reads 18 to 41-nt in length were aligned to 210-bp *dvssj1* sequence. Only perfectly matched reads were included for analysis of sequence length distribution of siRNA. The 18- and 41-nt *dvssj1* reads were visualized using Integrative Genomics Viewer software (Broad Institute, Cambridge, MA, USA) or aligned to 210 bp *dvssj1* using Sequencher (v 4.8, Gene Codes Corporation) to identify siRNA (>20 counts) regions (S5 Fig).

### RNA binding to midgut cells

Fifty diet-raised 2^nd^ instar WCR were treated and surface-sterilized as previously described [15], with modifications (S1 Method). Twenty midguts were dissected and rinsed in sterile 1x PBS to eliminate gut contents. Pretreated midguts were incubated with Cy3-labeled *dvssj1* dsRNA and siRNAs at 10 ng/μl in insect medium (EX-CELL 420 Serum-Free Medium; Sigma) for 15 hours at 25°C protected from light. Unconjugated Cy3 dye was used as a control at the same concentration. Midguts were washed twice with 1x PBS and fixed in 4% paraformaldehyde for 1 hour at room temp. Then midguts were washed three times with 1x PBST (PBS +0.1% Tween) for 5 minutes each. Samples were counterstained with DAPI (Sigma, 10 μg/μl of stock diluted to 1:1000) for 5 min and washed once in 1x PBST. Individual guts were placed on glass slides with 2x SlowFade concentrated Slow Fade antifade reagent in PBS, then coverslipped and imaged on a Leica TCS SPE. The 405 nm laser line was used for excitation of DAPI staining and the 532 nm laser line was used for excitation of Cy3 staining. Image analyses were carried out as described in S1 Method.

### Double-stranded RNA treatment for target suppression analyses

To evaluate SSJ protein and mRNA expression after *dvssj1* dsRNA treatments, 4-day old larvae were transferred to 96-well plate containing 167 ng/μl of dvssj1 dsRNA (ds*ssj1*) or *gfp* dsRNA (ds*gfp*) in 30 μl of an artificial diet [17] for 48 hours. Treated larvae were then transferred to the new plates containing 167 ng/μl of ds*gfp* for an additional five days. The larvae were then harvested for IHC and ISH microscopic studies as described in S1 Method and S12 and S13 Figs.

### Generation of *D*. *melanogaster ssk* knockout and *dvssj1* replacement lines via CRISPER/Cas9

CRISPR/Cas9-mediated genome editing by homology-dependent repair (HDR) using two guide RNAs and a dsDNA plasmid donor was used for replacing *ssk* with homolog *dvssj1* or making an ssk deletion mutant at the same breakpoint. The ScarlessDsRed system [32, 33] was employed to facilitate genetic screening. Edited *ssk* deletion and *dvssj1*-replacing lines were generated and confirmed by WellGenetics Inc (Taiwan) as described in S1 Method and S9, S10, S11 Figs and S2 Table. Since *ssk* is a lethal gene, edited lines were maintained as heterozygous in TM6B (containing visual marker). Crossing experiments before and after excision of DsRed were conducted for *dvssj1* or *ssk* viability evaluation.

## Results and discussion

### DVSSJ1 interacts with other SSJ proteins for essential midgut functions

The insect midgut is the primary organ for digestion and nutrient absorption [34]. The midgut epithelium mediates nutrient uptake and serves as the barrier separating the extracellular fluid of the body from the gut lumen and thereby protects the body from toxic substances and infectious organisms present in the environment. We previously reported the discovery of the *dvssj1* gene through dsRNA diet-based screening for targets that have the potential for WCR control [17]. However, the gene function was unknown at the time of our discovery and remained so until the report that its *D*. *melanogaster* ortholog, *ssk*, is required for intestinal barrier function by contributing to SSJ formation [21, 22]. The SSJ-specific membrane proteins, Ssk and Mesh, were identified by screening monoclonal antibodies raised against SSJ-containing membrane fractions[23, 24]. Recently, a genetic screen based on microscopic observation of SSJ formation in *D*. *melanogaster* identified Tsp2A (a homolog of *dvssj3*; S1 Fig) as a novel SSJ-specific membrane protein [25]. All three *D*. *melanogaster* SSJs form a protein complex that contributes to the specialization between epithelial cell apical and basolateral membranes and are required for *D*. *melanogaster* SSJ formation and intestinal barrier function [21].

### Midgut-specific expression of *dvssj1* during different life stages

**Fig 1:**
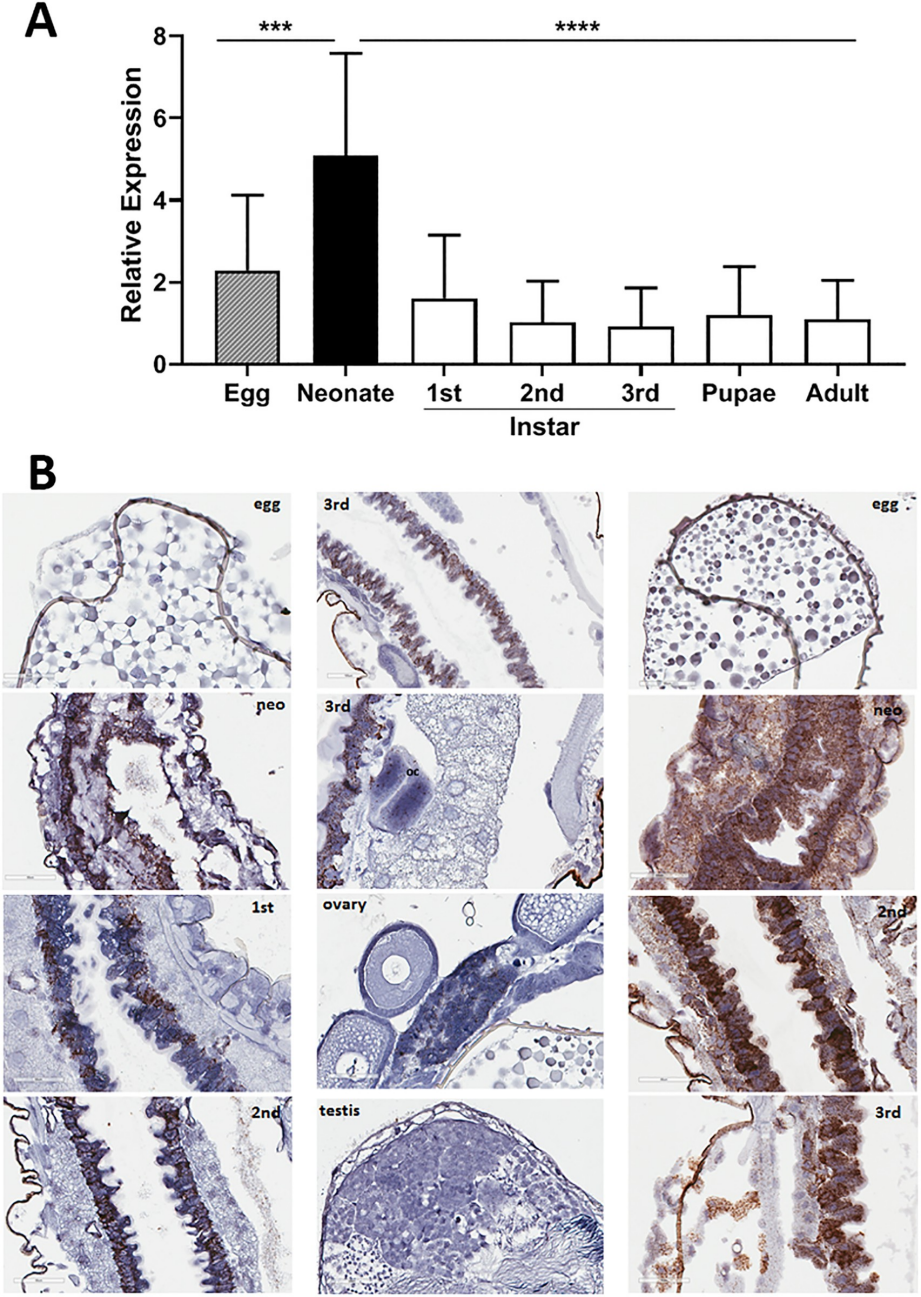
Expression and localization of *dvssj1* mRNA at different life stages of western corn rootworm (WCR). (A) Relative expression of the whole insect of WCR was measured at different life stages by qRT-PCR. Relative expression analysis (mean ± SE) was based on *dvssj1* expression in individual insects (n = 12) at each life stage, after being normalized to the expression of the reference gene, *dvrps10*. Expression data were subjected to one-way analysis of variance using JMP (v12. SAS Institute Inc, Cary, NC) followed by Tukey’s multiple comparison tests. Neonate stage showed statistically significant high expression (P = 0.004[***] or < 0.0001[****]). (B) Visualization of *dvssj1* mRNA expression during different life stages by *in situ* hybridization (ISH). Representative *WCR* sections were collected from the egg, neonate (neo; first 24-hrs after hatch), 1^st^, 2^nd^ and 3^rd^ instar of larvae, and dissected testis and ovary from adults. All samples were hybridized with the *dvssj1* probe and a control probe (*dvrps10*) were included for egg, neonate, 2^nd^ and 3^rd^ instars to compare (S2 Fig). Images were captured at 40x magnification with 60 μm scale bars.

Expression of *dvssj1* mRNA was analyzed by real-time quantitative reverse transcription PCR (qRT-PCR) using individual whole insects representing different life stages (Fig 1A), and by *in situ* hybridization (ISH) analysis of specific life stages, and dissected reproductive tissues from the WCR adults (Fig 1B, S2 Fig). The mRNA expression of *dvssj1* showed clear differences depending on the life stage. For example, *dvssj1* mRNA expression was about 4- or 5-fold higher in newly hatched neonates than in larvae, pupae, or adults. Localization of *dvssj1* mRNA occurred predominantly in the cells of the midgut epithelium (Fig 1B) during different stages of larval development which was in contrast to ribosomal protein s10 (*dvrps10)*[17], which was highly expressed in every visible cell type (Fig 1B). Expression of *dvssj1* mRNA in WCR has also been detected in oenocyte cells [35] and cells within Malpighian tubules (MTs) (Fig 1B) which are thin fingerlike extensions attached to the exterior intestinal tract between the midgut and the posterior gut or hindgut [36, 37].

**Fig 2:**
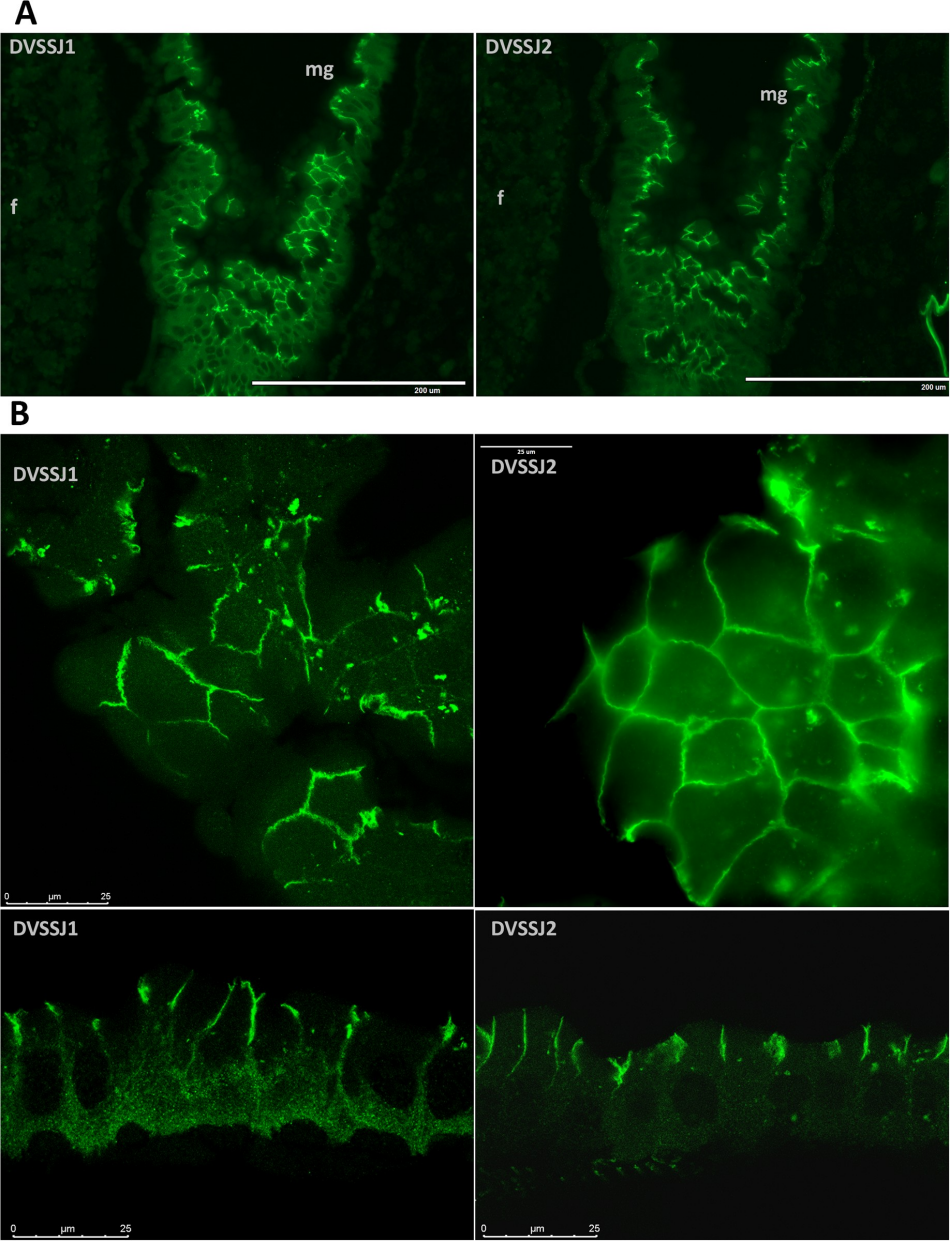
Immunofluorescence microscopic analysis of DVSSJ1 and DVSSJ2 in WCR larvae. (A) two adjacent sections were selected to compare DVSSJ 1 (left) and DVSSJ2 (right) expression. Images are representative sections (S3 Fig) of midguts (mg) collected from the 3^rd^ instar of WCR larvae and hybridized with antibodies as described in Method. (B) confocal images of selected midgut cells showing DVSSJ1 and DVSSJ2 localization. Scale bar = 200 um (A) and bar = 25 um (B).

WCR SSJ proteins were visualized by immunohistochemistry (IHC) using both peptide (DVSSJ2)[17] and total protein (DVSSJ1) antibodies as described in the S1 Method. Expression of DVSSJ1 and DVSSJ2 in the midgut (Fig 2) was observed in the longitudinal sections of whole larvae showing patterns that were reminiscent of the *D*. *melanogaster* orthologs snakeskin [23] and mesh [24]. The strongest immunostainings of SSJ1 or SSJ2 in midgut were observed at the apico-lateral part of epithelial column cells (Fig 2B), while weak or no signal was found in the foregut or hindgut (S3 and S4 Figs). Neither SSJ protein was observed in fat body cells, but consistent with their mRNA expression patterns they were detected in SSJs in oenocytes [35] and MTs (S4 Fig), which are responsible for lipid processing and detoxification [38], or excretory and osmoregulatory functions [39, 40], respectively. In *D*. *melanogaster*, SSK expression appears at stage 12 embryos in midgut rudiments and its expression is sustained until the adult stage throughout the midgut and MTs [23].

### Dose-dependent effects of dsRNA exposure are correlated to target suppression, larval development and survival

**Fig 3:**
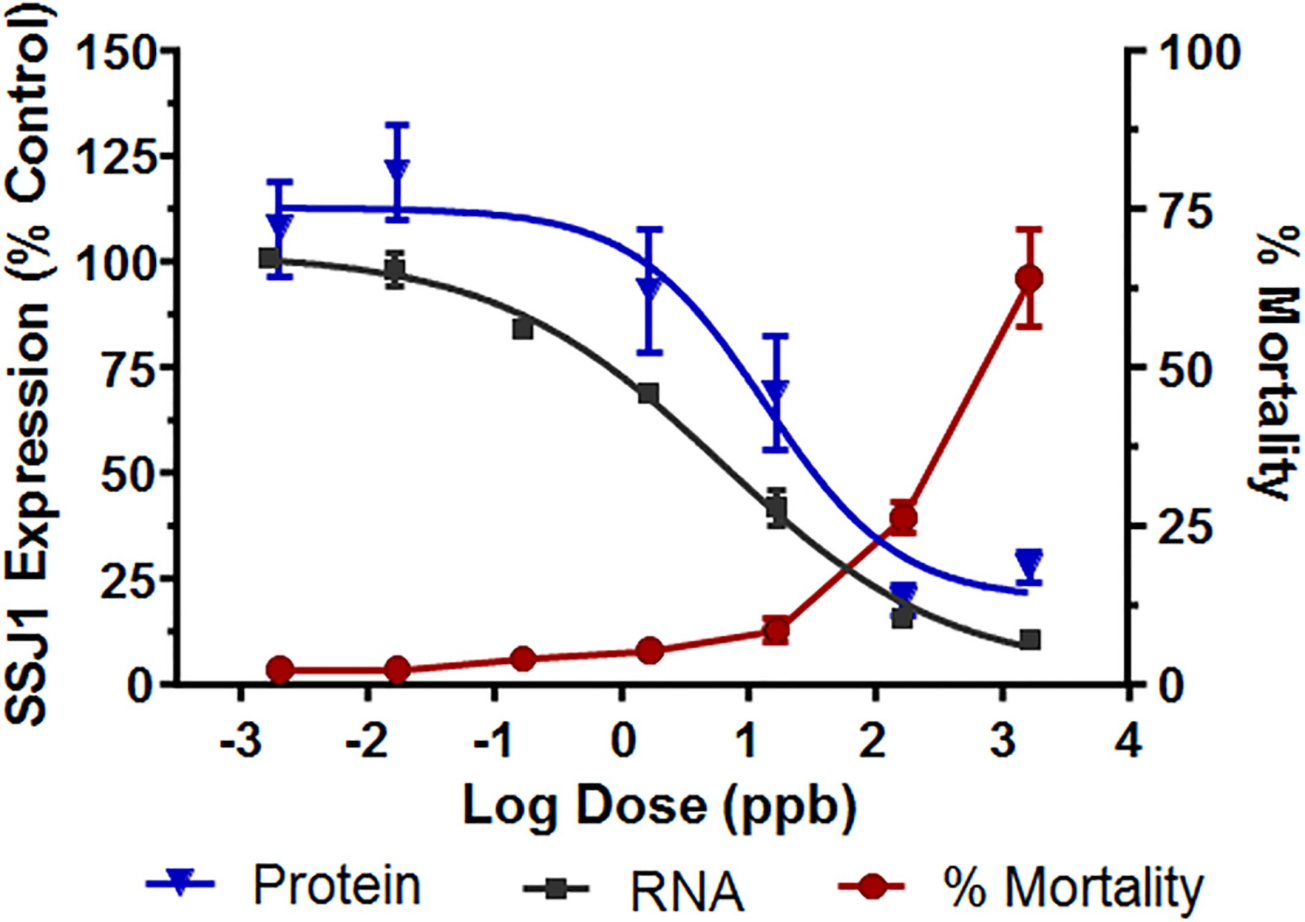
*Dvssj1* transcript and protein suppression correlate to dsRNA dose, as measured two days post-exposure. Larval mortality correlates with the extent of suppression, as measured seven days post-exposure. Larvae were infested on artificial diet alone, or with GUS dsRNA, or with increasing concentrations of *dvssj1* dsRNA as described in methods. After 48 hours feeding, larvae were collected and processed as described in order to assess the expression of the *dvssj1* transcript (black squares) or protein (blue triangles). Curves fit to the data were generated using a square logistic equation as described in methods. Duplicate sister plates were infested in order to follow larval growth inhibition (see S6 Fig) and mortality (red) for five additional days allowing the impact caused by the dose-dependent suppression of *dvssj1* transcript and protein expression to be quantified. The data reflect 3 independent determinations. Errors bars for each curve are explained in methods.

To examine the relationship between exposure to *dvssj1* dsRNA and suppression of *dvssj1* transcript and protein levels, WCR neonates were provided diet containing a range of *dvssj1* dsRNA concentrations. Larvae were assessed for survival and extent of growth and developmental inhibition after two and seven days of exposure. Larval development and mortality were consistent across all treatments and not different from controls after two days, whereas nearly all larvae showed growth inhibition or mortality after seven days exposure to the highest dsRNA dose (S6 Fig). As expected, the mortality and growth inhibition observed at seven days increased with increasing dsRNA dose (S5B Fig). To minimize the impact of natural variability in development and mortality between treatment and control groups, *dvssj1* transcript and protein levels were measured after two days of exposure. These results revealed a direct correlation between the dose of *dvssj1* dsRNA and suppression of mRNA, protein, and the mortality observed after 7-day treatments (S7 Fig). Fitting of the data revealed 50% suppression of transcript and protein at approximately 6.1×10^−2^ ng/μl and 1.4×10^−2^ ng/μl, respectively, with less than 50% mortality at these concentrations observed at the end of seven days exposure (Fig 3 and S7 Fig).

*Dvssj1* mRNA expression patterns (Fig 1B) and *dvssj1* mRNA knockdown (S12C Fig) were also demonstrated in WCR larvae using RNAscope ISH. Both mRNA and protein of *dvssj1* were observed predominantly in midgut epithelial cells and expression patterns varied slightly between different regions of the midgut (S2, S3 and S4 Figs). Similar expression patterns were also observed in guts dissected from WCR larvae (S5 Fig). The gene expression of *dvssj1* mRNA in midgut epithelial cells of WCR is consistent with a functional role of *dvssj1* analogous to *D*. *melanogaster ssk* [23]. Microscopic observations of nearly whole insects in section (S12C Fig) showed a significant difference in the overall size of *dvssj1*-treated and control individuals. Previous ultrastructural examination of midgut epithelial cells revealed apparent occlusion of the gut lumen, and numerous examples of enterocytes blebbing into the gut lumen, after *dvssj1* dsRNA consumption [17]. These observations are consistent with the notion that suppression of *dvssj1* mRNA and its protein accumulation are the cause of WCR growth inhibition and mortality. Furthermore, the toxic effect to WCR resulting from oral exposure to *dvssj1* dsRNA in diet or expressed *in planta* [17] can be attributed to suppression of *dvssj1* mRNA leading to a reduction in DVSSJ1 expression/accumulation, loss of the midgut epithelium diffusional barrier, and cellular deformities due to improper intercellular contacts [22].

### Double-strand RNA processing and uptake in WCR midgut

**Fig 4:**
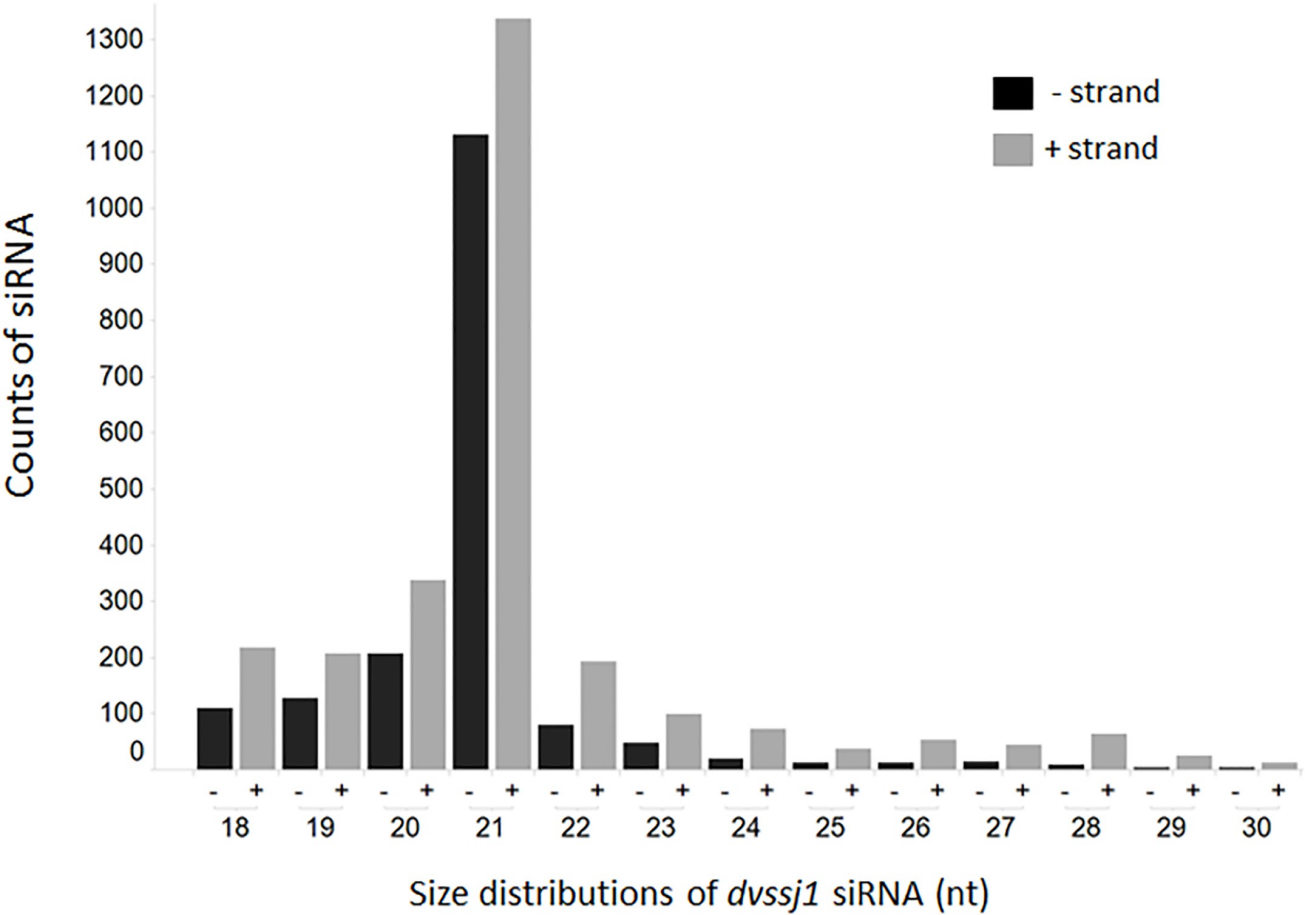
Size distribution of *dvssj1* small RNAs between 18 and 30 nucleotides. Small RNA sequencing of WCR larvae fed with 210-bp *dvssj1* dsRNA was conducted to identify siRNAs mapped to 210-bp region of *dvssj1*. Sense (grey) and antisense (black) siRNA distribution was compared between 18 and 30 nucleotides.

To understand dsRNA processing in WCR midgut, we performed an RNAseq study to characterize small RNA generated from 3^rd^ instar larvae exposure to 210-bp dsRNA of *dvssj1*. The siRNA sequences mapped to the sense and antisense strands of the 210-bp *dvssj1* sequence (Fig 4 and S8 Fig). These small RNAs are distributed across the 210 bp-length of the *dvssj1* sequence, with three regions that have dominant siRNA accumulation over other regions (S8 Fig). The top siRNA represented 14.1% of all *dvssj1* siRNA (Table 1).

**Table 1:** Summary of small RNAs (18 to 41 nt) mapped to 210 bp of *dvssj1*.

siRNA summary	Count
total count of all *dvssj1* siRNA	4543
total number of siRNA type	818
number of minus strand	1790
number of plus strand	2751
siRNA with above 20 counts*	
region-1 (32-76nt)	488
region-2 (93-132nt)	1538
region-3 (169-191nt)	170
**Top siRNA in region-2**	
TCCTTGATATCCGGTTCGGTA	641

*Three regions contain small RNAs with more than 20 counts were grouped based on their mapped locations to 210 bp of *dvssj1* (S8 Fig).

**Fig 5:**
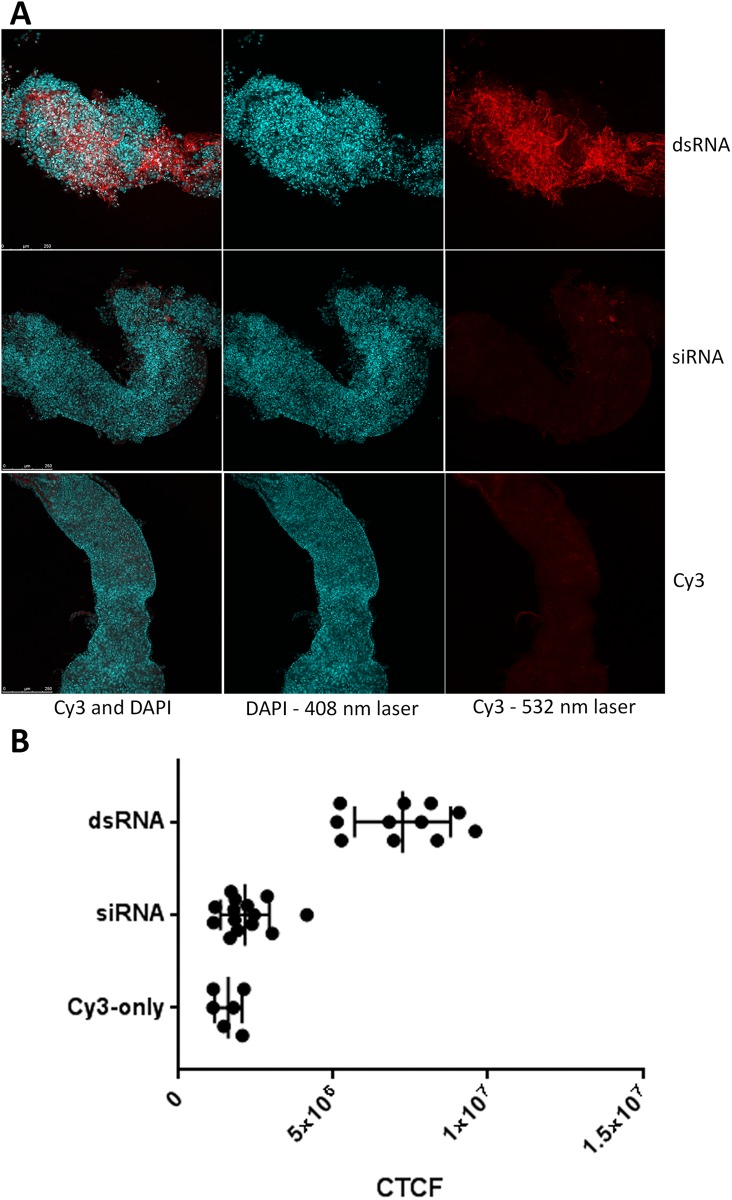
RNA binding to midgut cells of western corn rootworm (WCR). (A) Cy3-labeled 210 *dvssj1* dsRNA is binding to WCR midgut cells (Top panel), but 21-bp siRNA of *dvssj1* is not observed (middle panel). Commercial Cy3 dye was used as a control, which showed no binding also. (B) Summary of the fluorescent intensity of midgut cells. Corrected Total Cell Fluorescence (CTCF) was calculated in ImageJ. DsRNA treatment (n = 11) is significantly different (P<0.0001) from control and siRNA treatment (n = 14).

To evaluate dsRNA uptake in WCR midgut, Cy3-labelled 210 bp *dvssj1* dsRNA and Cy3-labelled siRNA (21-mer) representing one of the top siRNAs were incubated with dissected WCR midgut tissues. The 210 bp Cy3-dsRNA was able to clearly bind to midgut cells, while in contrast, Cy3-siRNA did not bind to the midgut as determined by the absence of a detectable Cy3 label (Fig 5A). Controls with insect medium containing unincorporated Cy3 dye showed no fluorescence binding to the midgut (Fig 5A). Two independent experiments were conducted and a summary of results shown in Fig 5B. These results under test conditions are consistent with previous reports of dsRNA uptake and ineffectiveness of exogenous siRNA (21-mer) binding to WCR midgut [15].

**Fig 6:**
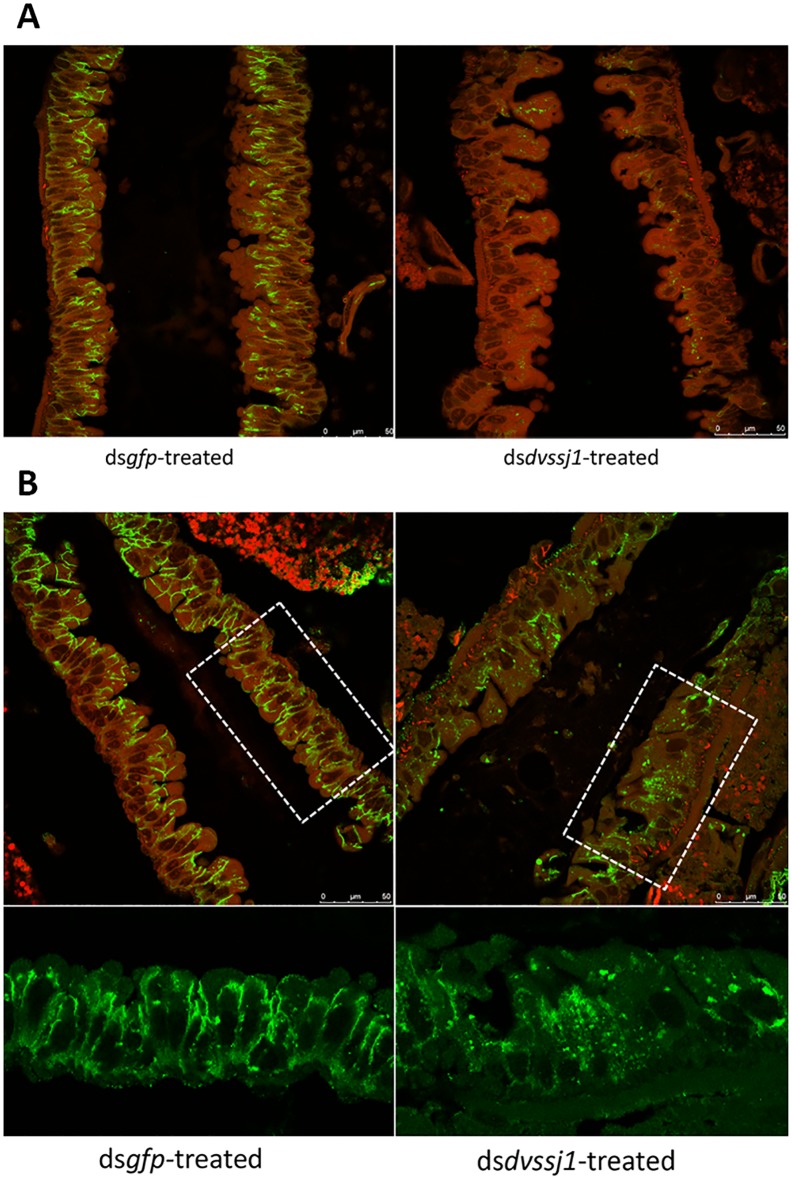
Immunohistochemistry detection of SSJ proteins in dsRNA-treated midgut cells. WCR larvae (4-day old) were treated with 167 ng/μl dsRNA in the diet for 48 hours (*gfp* left panel and *dvssj1* right panel), and collected 7 days after treatment, and hybridized with the DVSSJ1 (A) and DVSSJ2 (B) antibodies. Confocal images were captured with combined GFP and DAPI channels. Boxed areas were enlarged to show DVSSJ2 expression pattern changes (GFP channel only) after *dvssj1* dsRNA treatment. Scale bar = 25 μm.

Since molecular characterizations of SSJ has been reported in *D*. *melanogaster* [23] and technical challenges have hindered conducting similar midgut barrier functional evaluations in WCR, we generated *D*. *melanogaster ssk* knock-out and *dvssj1* knock-in substitution (S9, S10 and S11 Figs) lines using CRISPR-Cas9-mediated genome editing. As expected, homozygous *ssk* knockout resulted in a lethal phenotype at the larval stage (Table 2). Molecular substitution of *ssk* by *dvssj1* resulted in only survival of heterozygous *dvssj1* knock-ins suggesting that there is insufficient homology (54.9%) of the WCR protein [17] to allow interaction with other fly SSJ components and substitute in SSJ formation (Table 2 and S11 Fig). Furthermore, it has been reported that *D*. *melanogaster* SSK interacts with MESH for midgut ba**r**rier functions and these proteins are mutually interdependent for their localization [24]. Examination of SSJ protein expression in WCR midgut cells after *dvssj1* dsRNA-tr**e**atment showed clear DVSSJ1 protein knockdown and the consequent mislocalization of DVSSJ2 protein (Fig 6). We previously reported that suppression of *dvssj2* [17] and *dvssj3* [41] expression can cause WCR mortality in artificial diet delivery bioassays. These results indicate that WCR SSJ proteins have a similar function like *D*. *melanogaster* SSJs, contributing to a protein complex that is required for SSJ formation and normal intestinal barrier function [22].

**Table 2:** Summary of viability test for edited *ssk*-*D*. *melanogaster* lines

Edited line	*dvssj1* knockin	*ssk* deletion
Before DsRed excision		
Heterozygous	207	216
Homozygous	0	0
After DsRed excision		
Heterozygous	289	241
Homozygous	0	0

Two separate viability tests were conducted before and after DsRed maker excision. Both Drosophila *ssk* (deletion) knock-out and *dvssj1* knock-in substitution lines were crossed and maintained in the balance line (S1 Method and S10 Fig). A number of survival insects were counted based on Tubby phenotype (TM6B). No homozygous edited fly was recovered.

## Conclusion

Our findings show that disruption of SSJ function by down-regulation of *dvssj1* expression can be correlated directly with insect mortality making it a well-suited gene target for RNAi silencing and providing an alternative molecular target for control of corn rootworm. This study also illustrates that *dvssj1* is a midgut-specific gene in WCR and its functions are consistent with biological functions described for *ssk* in Drosophila.

## Supporting information

S1 Fig Sequence alignment of WCR SSJ3 and *D*. *melanogaster* Tsp2A. (DOCX)

S2 Fig Analyses of *dvssj1* mRNA expression during different life stages of *Diabrotica virgifera virgifera* by *in situ* hybridization (ISH). (DOCX)

S3 Fig Immunohistochemistry (IHC) detection of DVSSJ1 and DVSSJ2 proteins. (DOCX)

S4 Fig Immunohistochemistry detection of DVSSJ1 and DVSSJ2 in gut tissues. (DOCX)

S5 Fig Expression of *dvssj1* mRNA and protein in WCR first through third instar larval tissues. (DOCX)

S6 Fig Average larval distribution and mortality observed 2-day and 7-day post-*dvssj1* dsRNA exposure (ppb or pg/μl). (DOCX)

S7 Fig The dose relationships for transcript and protein expression. (DOCX)

S8 Fig Identification of *dvssj1* siRNAs in 3^rd^ instar fed with dsRNA of *dvssj1* frag1. (DOCX)

S9 Fig Targeting *D*. *melanogaster ssk*/CG6981 *via* CRISPR/Cas9-mediated genome editing. (DOCX)

S10 Fig PCR confirmation of edited lines after DsRed excision. (DOCX)

S11 Fig Confirmation of edited lines by sequencing PCR products. (DOCX)

S12 Fig Slide images of dsRNA-treated larvae used for immunohistochemistry (IHC) and *in situ* hybridization (ISH). (DOCX)

S13 Fig Representative images of DVSSJ1 and DVSSJ2 expression after dsRNA treatment. (DOCX)

S1 Method Antibody preparation; optimization for immunohistochemistry (IHC); RNA binding to midgut cells; *Drosophila melanogaster ssk* knockout and knockin line *via* CRISPER/Cas 9. (DOCX)

S1 Table Primers, oligos and antibody information. (DOCX)

S2 Table CRISPER-CAS9 target sites, primer (oligo), and guide RNAs. (DOCX)
