# Supplementary material for: Molecular characterization of the insecticidal activity of double-stranded RNA targeting the smooth septate junction of western corn rootworm (Diabrotica virgifera virgifera)
Source: PLoS One. 2019 Jan 10;14(1):e0210491. doi: 10.1371/journal.pone.0210491 (PMC6328145; doi:10.1371/journal.pone.0210491)
Supplement: S1 Fig — (DOCX) [file pone.0210491.s001.docx]

1.........11........21........31........41........51.......60

Dv-ssj3 MMSKVDTQMMSKADTQEDASFAKLENQIAIIKYVILFTNVLQWALGAAIFALCLWLRFEE

Dv-ssj3b --------MMSKADTQEDASFAKLENQIAIIKYVILFTNVLQWALGAAIFALCLWLRFEE

Tsp2A-PA ------------MGIGYGASDEQLEKQIGCVKYTLFCFNIVAWMISTALFALTVWLRAEP

Tsp2A-PB ------------MGIGYGASDEQLEKQIGCVKYTLFCFNIVAWMISTALFALTVWLRAEP

. .** :**:**. :**.:: *:: * :.:*:*** :*** *

61........71........81........91........101.......111......120

Dv-ssj3 GIQEWLQKLDSEQFYIGVYVLIVASLIVMIVSFIGCISALQESTMALLVYIGTQVLSFIF

Dv-ssj3b GIQEWLQKLDSEQFYIGVYVLIVASLIVMIVSFIGCISALQESTMALLVYIGTQVLSFIF

Tsp2A-PA GFNDWLRILEAQSFYIGVYVLIGISIVMMAVSFLGCLSALMENTLALFVFVGTQVFGFIA

Tsp2A-PB GFNDWLRILEAQSFYIGVYVLIGISIVMMAVSFLGCLSALMENTLALFVFVGTQVFGFIA

*:::**: *:::.********* *:::* ***:**:*** *.* **:*::****:.**

121.......131.......141.......151.......161.......171......180

Dv-ssj3 GLSGSAVLLDNSARDSHFQPRIRESMRRLIMNAHHDQSRQTLAMIQENVGCCGADGATDY

Dv-ssj3b GLSGSAVLLDNSARDSHFQPRIRESMRRLIMNAHHDQSRQTLAMIQENVGCCGADGATDY

Tsp2A-PA IVAGSAVLLQFSTINSSLQPLLNVSLRGFVATSEYTYSNYVLTMIQENIGCCGATGPWDY

Tsp2A-PB IVAGSAVLLQFSTINSSLQPLLNVSLRGFVATSEYTYSNYVLTMIQENIGCCGATGPWDY

::******: *: :* :** :. *:* :: .:.: *. .*:*****:***** *. **

181.......191.......201.......211.......221.......231......240

Dv-ssj3 LSLQQPLPSQCRDTVTGNPFFHGCVDELTWFFEEKCGWIAGLAMAICMINVLSIVLSTVL

Dv-ssj3b LSLQQPLPSQCRDTVTGNPFFHGCVDELTWFFEEKCGWIAGLAMAICMINVLSIVLSTVL

Tsp2A-PA LDLRQPLPSSCRDTVSGNAFFNGCVDELTWFFEGKTGWIVALAMTLGLLNVICAVMSFVL

Tsp2A-PB LDLRQPLPSSCRDTVSGNAFFNGCVDELTWFFEGKTGWIVALAMTLGLLNVICAVMSFVL

* *:*****.*****:**.**:*********** * ***..***:: ::**:. *:* **

241.......251..256

Dv-ssj3 IQALKKEEEASDSYRR

Dv-ssj3b IQALKKEEEASDSYRR

Tsp2A-PA VQAVKKEEEQASNYRR

Tsp2A-PB VQAVKKEEEQASNYRR

:**:***** :..***

**S1 Fig. Sequence alignment of WCR SSJ3 and *D. melanogaster* Tsp2A.**

This alignment was derived using CLUSTAL W with default parameters[6]. * (asterisk) represents identical amino acid residues shared between *dvssj3* and *Tsp2A*, : (colon) conservation between two amino acid residues of strongly similar properties and . (period) indicates conservation between two amino acid residues of weakly similar properties. International Patent Application No WO 2017/218207. 2017.Bottom of Form
