# Supplementary material for: Molecular characterization of the insecticidal activity of double-stranded RNA targeting the smooth septate junction of western corn rootworm (Diabrotica virgifera virgifera)
Source: PLoS One. 2019 Jan 10;14(1):e0210491. doi: 10.1371/journal.pone.0210491 (PMC6328145; doi:10.1371/journal.pone.0210491)
Supplement: S2 Fig — (DOCX) [file pone.0210491.s002.docx]

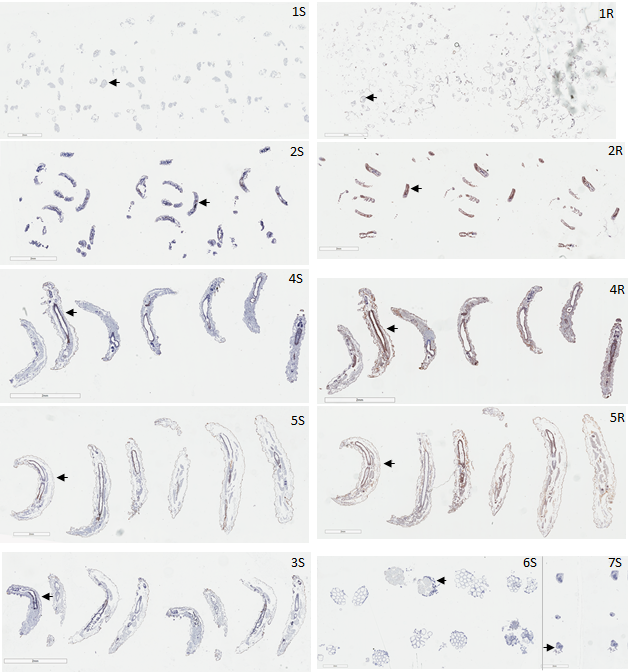


**S2 Fig. Analyses of *dvssj1* mRNA expression during different life stages of *Diabrotica virgifera virgifera* by *in situ* hybridization (ISH).**

Multiple insects or dissected reproductive tissues were placed onto slides for *in situ hybridization* and only one or two objects (arrow) were selected for high-resolution images in Fig 1A. WCR samples were collected from the egg (1), neonate (2), 1^st^ (3), 2^nd^ (4) and 3rd instar (5) of larvae, dissected ovaries (6) and testis (7), and hybridized with the *dvssj1*(S) and *rps10* (R) probes. Adjacent slides of the same 2^nd^/3rd instar tissues were used for expression comparison with *dvrps10*. Slide images were adjusted to scale=2mm
