# Supplementary material for: Molecular characterization of the insecticidal activity of double-stranded RNA targeting the smooth septate junction of western corn rootworm (Diabrotica virgifera virgifera)
Source: PLoS One. 2019 Jan 10;14(1):e0210491. doi: 10.1371/journal.pone.0210491 (PMC6328145; doi:10.1371/journal.pone.0210491)
Supplement: S3 Fig — (DOCX) [file pone.0210491.s003.docx]

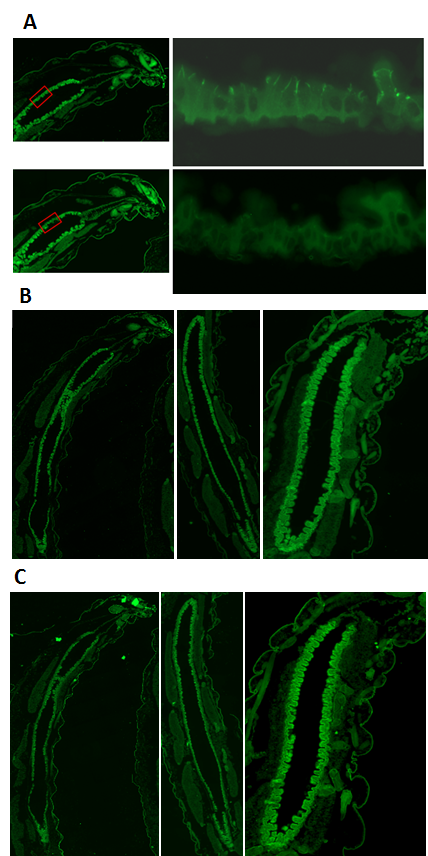


**S3 Fig. Immunohistochemistry (IHC) detection of DVSSJ1 and DVSSJ2 proteins.** (A) Adjacent 3^rd^ instar sessions containing gut tissues were placed onto slides for immunofluorescence microscopy to compare antibodies from total protein (top) and peptide (bottom). Representative gut images **of** DVSSJ1 (B) and DVSSJ2 (C) antibodies were prepared as described in Supporting Method. Three insects were selected for high-resolution images of gut tissues in Fig 1A and S4 Fig.
