# Supplementary material for: Molecular characterization of the insecticidal activity of double-stranded RNA targeting the smooth septate junction of western corn rootworm (Diabrotica virgifera virgifera)
Source: PLoS One. 2019 Jan 10;14(1):e0210491. doi: 10.1371/journal.pone.0210491 (PMC6328145; doi:10.1371/journal.pone.0210491)
Supplement: S4 Fig — (DOCX) [file pone.0210491.s004.docx]

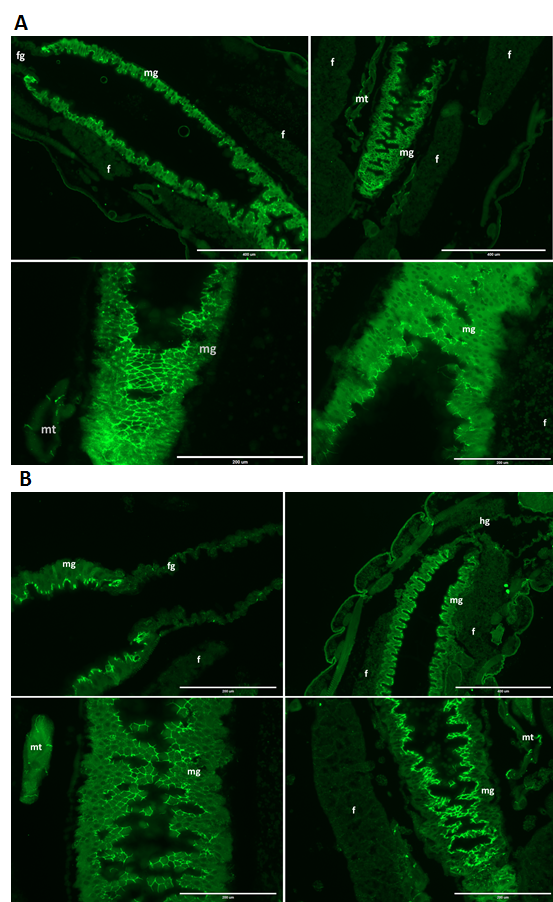


**S4 Fig. Immunohistochemistry detection of DVSSJ1 (A) and DVSSJ2 (B) in gut tissues.** Scale bar=200 µm; fg=foregut, mg=midgut, hg=hindgut, f=fat body, mt=malpighian tubules.
