# Supplementary material for: Molecular characterization of the insecticidal activity of double-stranded RNA targeting the smooth septate junction of western corn rootworm (Diabrotica virgifera virgifera)
Source: PLoS One. 2019 Jan 10;14(1):e0210491. doi: 10.1371/journal.pone.0210491 (PMC6328145; doi:10.1371/journal.pone.0210491)
Supplement: S5 Fig — (DOCX) [file pone.0210491.s005.docx]

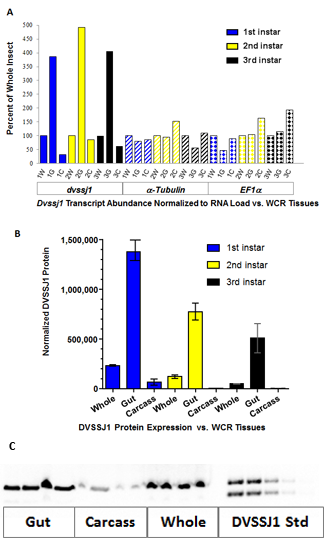


**S5 Fig. Expression of *dvssj1* mRNA (A) and protein (B) in WCR first through third instar larval tissues.** Panel A shows transcript abundance (relative to RNA load) of *dvssj1*, -*tubulin* and *EF1a* in whole (W), isolated gut (G), and carcass (C) after gut removal for first, second and third instar larvae normalized to the values measured for whole larvae. Panel B shows DVSSJ protein determined by band densitometry (see main methods) of western blot images normalized to the total protein in the tissue homogenate. A representative western blot (panel C) is from samples collected from first instar larvae is also shown. Protein samples equivalent to 10 individual guts, carcasses or whole larvae were loaded in four replicates and standard DVSSJ1 protein (Supporting Method) was loaded 4, 3, 2, 1 and 0.5 ng per lane (left to right). These data confirm the use of whole insect samples for expression analysis and gut samples for protein expression analysis should not give disparate results.
