# Supplementary material for: Molecular characterization of the insecticidal activity of double-stranded RNA targeting the smooth septate junction of western corn rootworm (Diabrotica virgifera virgifera)
Source: PLoS One. 2019 Jan 10;14(1):e0210491. doi: 10.1371/journal.pone.0210491 (PMC6328145; doi:10.1371/journal.pone.0210491)
Supplement: S6 Fig — (DOCX) [file pone.0210491.s006.docx]

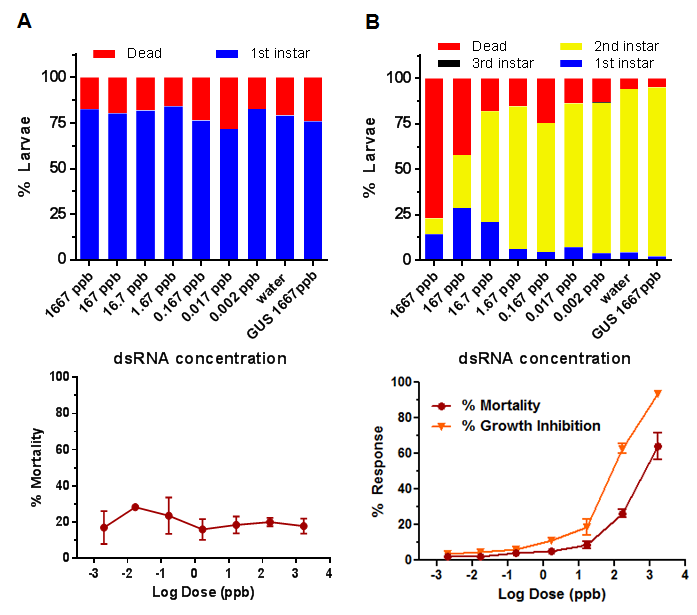


**S6 Fig. Average larval distribution and mortality observed 2-day (A) and 7-day (B) post-*dvssj1* dsRNA exposure (ppb or pg/µl).** (A) Upper: after 48 hours, some natural mortality was observed, but most larvae were 1^st^ instars as expected at this time point; lower: there was no dose effect on larval mortality indicated naturally occurring death (B) upper: After 7 days, the dose-dependent effects of the silencing by *dvssj1* dsRNA are obvious as increased mortality and growth inhibition was observed with increased dose; lower: growth inhibition shows the same dose-response trend that was observed for larval mortality also shown in Main Fig. 3. This combination of factors makes proper seven-day assessments of the *dvssj1* transcript and protein levels very difficult; a 48-hour exposure window is more consistent on all metrics, including stability of dsRNA.
