# Supplementary material for: Molecular characterization of the insecticidal activity of double-stranded RNA targeting the smooth septate junction of western corn rootworm (Diabrotica virgifera virgifera)
Source: PLoS One. 2019 Jan 10;14(1):e0210491. doi: 10.1371/journal.pone.0210491 (PMC6328145; doi:10.1371/journal.pone.0210491)
Supplement: S7 Fig — (DOCX) [file pone.0210491.s007.docx]

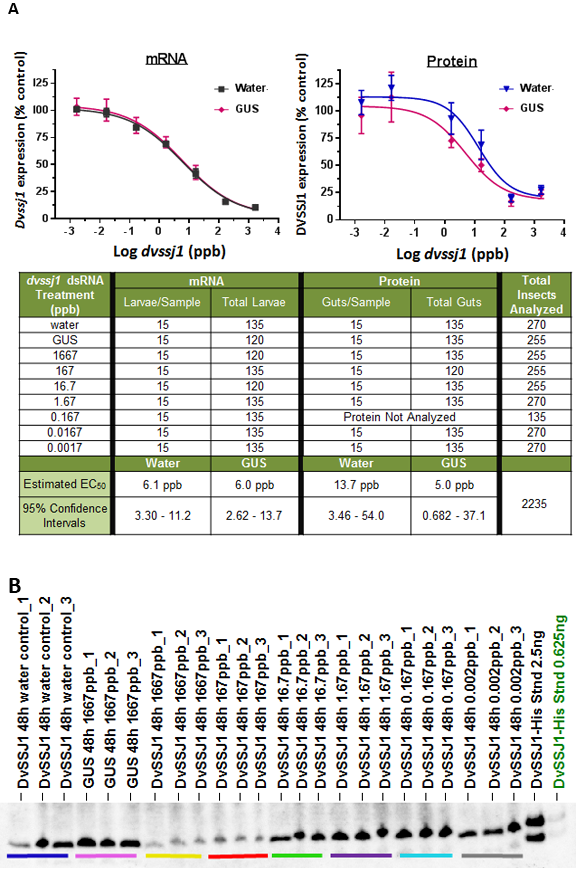


**S7 Fig. The dose relationships for transcript and protein expression showed similar trends regardless of which control treatments were used for normalization (water or 1667 ppb (pg/µl) GUS dsRNA).** (A) dose-response of the *dvssj1* transcript and protein expression two days’ post-exposure to seven treatments of *dvssj1* dsRNA, normalized to two control treatments (water or 1667 ppb GUS dsRNA) over three replicate assays. The control used for normalization does not affect results. Breakdown of sample processing for each treatment for each analysis (middle table). The EC_50_ values (half maximal effective concentration) from the logistic fits to the data in the dose-response curves are also provided for each method of normalization. There was an excellent agreement between the EC_50_ for suppressing *dvssj1* transcript and protein with half-effective concentrations ranging 5-10 ppb. These values also show nice overlap with the percent mortality shown in main Fig. 3 which is consistent with the role that DVSSJ1 protein plays in normal gut physiology necessary for insect viability. (B) Example of western blot analyses with three replicates.
