# Supplementary material for: Molecular characterization of the insecticidal activity of double-stranded RNA targeting the smooth septate junction of western corn rootworm (Diabrotica virgifera virgifera)
Source: PLoS One. 2019 Jan 10;14(1):e0210491. doi: 10.1371/journal.pone.0210491 (PMC6328145; doi:10.1371/journal.pone.0210491)
Supplement: S8 Fig — (DOCX) [file pone.0210491.s008.docx]

# **
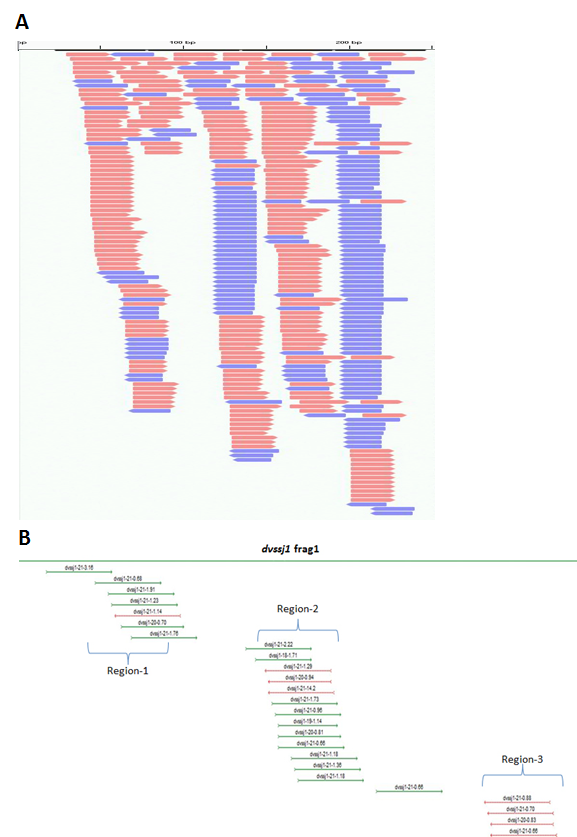
**

# **S8 Fig. Identification of *dvssj1* siRNAs in 3^rd^ instar fed with dsRNA of *dvssj1* frag1.**

# **(**A) siRNA profile of 3^rd^ instar larvae fed with 210 bp *dvssj1* dsRNA at 180 ng/µl in the diet for 48 hours. Sense (red) and antisense (blue) of siRNAs were aligned with 210 bp dsRNA *dvssj1* using integrative genomics viewer. (B) Alignment of individual siRNA (above 20 counts) with 210 bp *dvssj1*. Three regions of sense (green) and antisense (red) siRNAs were highlighted.
