# Supplementary material for: Molecular characterization of the insecticidal activity of double-stranded RNA targeting the smooth septate junction of western corn rootworm (Diabrotica virgifera virgifera)
Source: PLoS One. 2019 Jan 10;14(1):e0210491. doi: 10.1371/journal.pone.0210491 (PMC6328145; doi:10.1371/journal.pone.0210491)
Supplement: S9 Fig — (DOCX) [file pone.0210491.s009.docx]

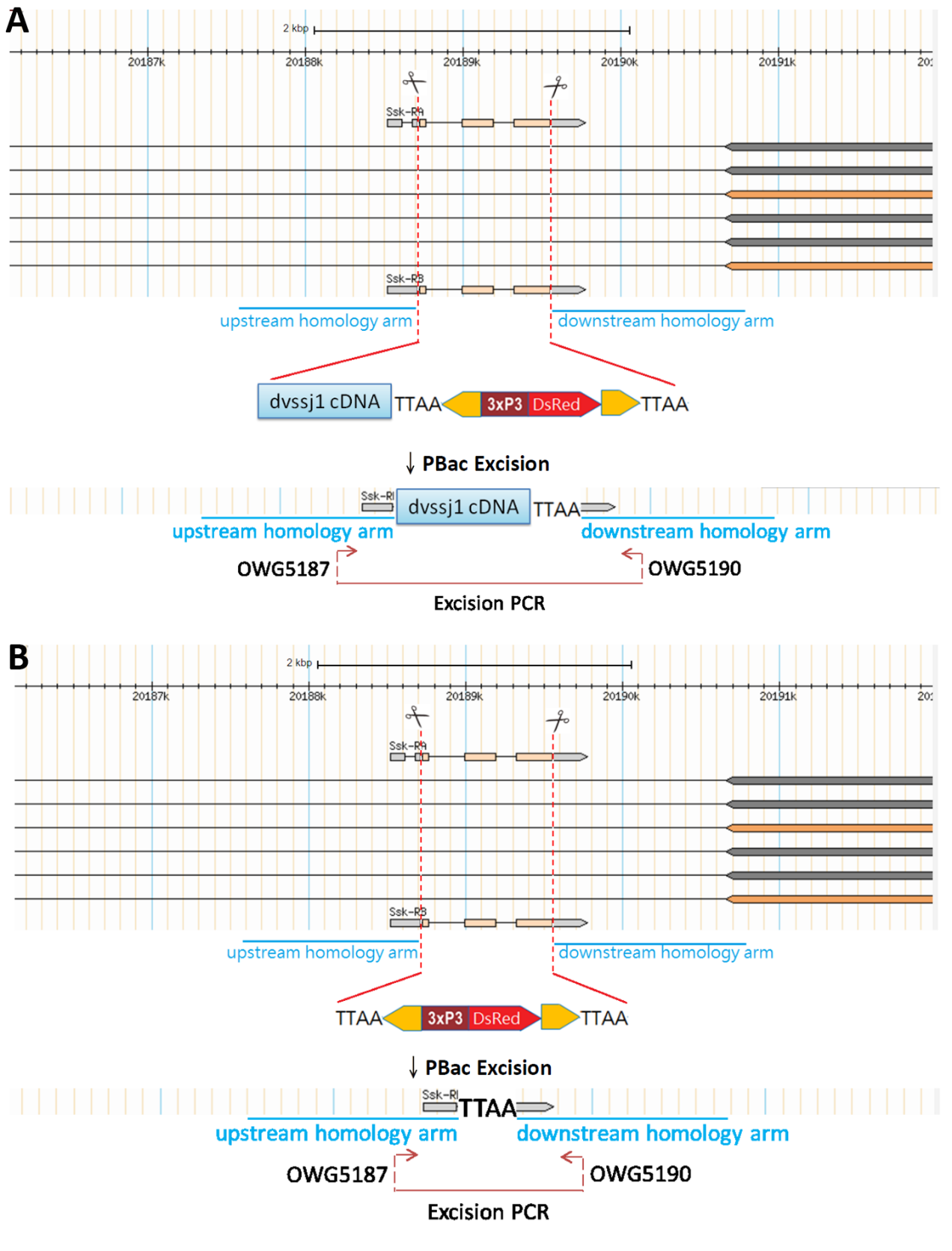


**S9 Fig. Targeting *D. melanogaster* *ssk*/CG6981 *via* CRISPR/Cas9-mediated genome editing**

(A) to replace *ssk* by homolog *dvssj1* and (B) to make *ssk* deletion mutant at the same break-point using the ScarlessDsRed system to facilitate genetic screening. Excision of selection marker (DsRed) was done by *PiggyBac* (PBac) transposition.
