# Supplementary material for: Molecular characterization of the insecticidal activity of double-stranded RNA targeting the smooth septate junction of western corn rootworm (Diabrotica virgifera virgifera)
Source: PLoS One. 2019 Jan 10;14(1):e0210491. doi: 10.1371/journal.pone.0210491 (PMC6328145; doi:10.1371/journal.pone.0210491)
Supplement: S10 Fig — (DOCX) [file pone.0210491.s010.docx]

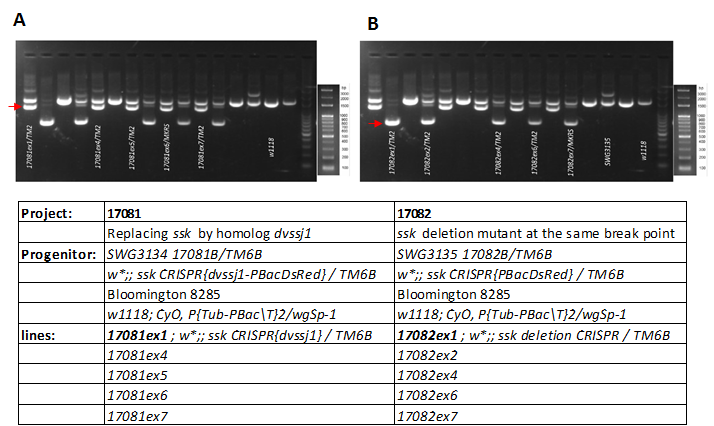


CRISPR/Cas9-mediated genome editing was used to target *ssk*/CG6981 by homology-dependent repair (HDR) using 2 guide RNAs and a dsDNA plasmid donor; the ScarlessDsRed system was used to facilitate genetic screening; Excision is validated by genomic PCR and sequencing. All positive lines showed homozygous lethal.

**S10 Fig. PCR confirmation of edited lines after DsRed excision.** PCR bands at expected size (red arrows) were observed from heterozygous samples of *17081ex1, 17081ex4, 17081ex5, 17081ex6* and *17081ex7* lines for Excision PCR (1153bp; A); as well as *17082ex1, 17082ex2, 17082ex4, 17082ex6* and *17082ex7* lines for Excision PCR (673bp; B). The wildtype PCR (1518bp) suggests that selection marker is excised. A larger band (2369bp) and wildtype (1518bp) band were shown in heterozygous progenitor, *SWG3135*. The wildtype band was observed in injection strain control, suggesting that high specificity of the PCR reaction. The PCR product (red arrow) of *17082ex1* (673bp) and *17081ex1* (1153bp) were cut and sent for sequencing. Genomic DNA was obtained from the single fly of each stock following single-fly DNA prep. Injection strain *w1118* was used as a negative control. PCR was performed using KOD-FX (TOYOBO) on BioRad S1000 Thermal Cycler. 100bp DNA Ladder from GenePure was used as a reference. Forward primer (OWG5187;5’-TTTCGAACGGCACCTGTTAT) is designed at upstream homology arm and reverse primer (OWG5190;5’-CTGAGCAACACGATCAATGG) is designed at downstream homology arm as illustrated above.
