# Supplementary material for: Molecular characterization of the insecticidal activity of double-stranded RNA targeting the smooth septate junction of western corn rootworm (Diabrotica virgifera virgifera)
Source: PLoS One. 2019 Jan 10;14(1):e0210491. doi: 10.1371/journal.pone.0210491 (PMC6328145; doi:10.1371/journal.pone.0210491)
Supplement: S11 Fig — (DOCX) [file pone.0210491.s011.docx]

**
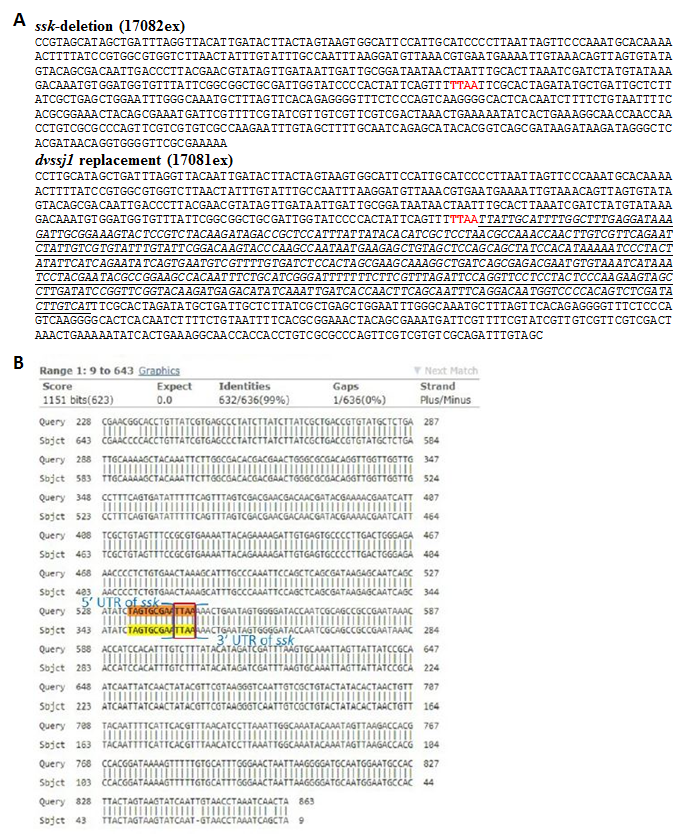
**

**S11 Fig. Confirmation of edited lines by sequencing PCR products.** (A) Sequences of PCR products *ssk*-deletion and *dvssj1* replacement lines. The exogenous TTAA motif was highlighted in red and *dvssj1* (reverse orientation) was marked by underline. (B) Sequence read (17082ex, Sbjct) was aligned with 17082 excised donor sequence (Query) using Blast2. One exogenous TTAA motif (boxed in red) was left between 5’ UTR and 3’ UTR of *ssk* (bracket in blue) in 17082ex.
