# Supplementary material for: Molecular characterization of the insecticidal activity of double-stranded RNA targeting the smooth septate junction of western corn rootworm (Diabrotica virgifera virgifera)
Source: PLoS One. 2019 Jan 10;14(1):e0210491. doi: 10.1371/journal.pone.0210491 (PMC6328145; doi:10.1371/journal.pone.0210491)
Supplement: S12 Fig — (DOCX) [file pone.0210491.s012.docx]

**
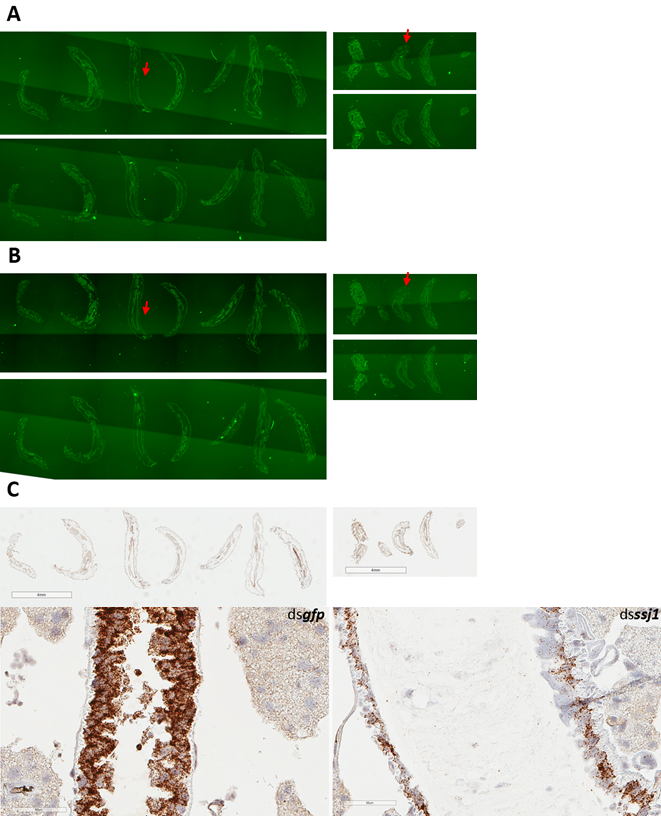
**

**S12 Fig. Slide images of dsRNA-treated larvae used for Immunohistochemistry (IHC) and *in situ* hybridization (ISH).** Multiple insects were placed onto slides for IHC (two sections) and ISH analyses. Selected insects (arrow) were selected for high-resolution images in Fig 6 or S13 Fig. WCR larvae (4-day old) were treated with 167 ng/µl dsRNA in diet for 48 hours (*gfp* left panel and *dvssj1* right panel*)*, and collected 7 days after treatment, and hybridized with the DVSSJ2 (A) and DVSSJ1 (B) antibodies or *dvssj1* RNA probes (C) as described in Method. Scale bar=4mm or 60 µm.
