# Supplementary material for: Molecular characterization of the insecticidal activity of double-stranded RNA targeting the smooth septate junction of western corn rootworm (Diabrotica virgifera virgifera)
Source: PLoS One. 2019 Jan 10;14(1):e0210491. doi: 10.1371/journal.pone.0210491 (PMC6328145; doi:10.1371/journal.pone.0210491)
Supplement: S13 Fig — (DOCX) [file pone.0210491.s013.docx]

**
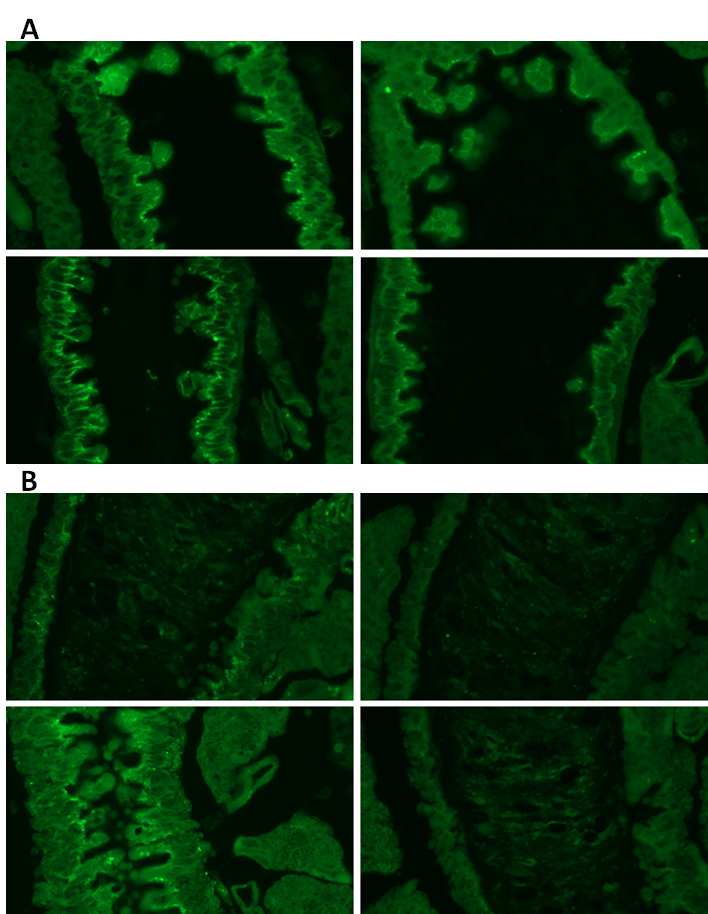
**

**S13 Fig. Representative images of DVSSJ1 and DVSSJ2 expression after dsRNA treatment. (**A) larvae treated with *gfp* dsRNA and hybridized with DVSSJ1 (right) and DVSSJ2 (left); (B) larvae treated with *dvssj1* dsRNA and hybridized with DVSSJ1 (right) and DVSSJ2 (left) antibodies.
