# Supplementary material for: Molecular characterization of the insecticidal activity of double-stranded RNA targeting the smooth septate junction of western corn rootworm (Diabrotica virgifera virgifera)
Source: PLoS One. 2019 Jan 10;14(1):e0210491. doi: 10.1371/journal.pone.0210491 (PMC6328145; doi:10.1371/journal.pone.0210491)
Supplement: S1 Method — (DOCX) [file pone.0210491.s014.docx]

# **S1 Method**

**Antibody preparation:** DVSSJ1 and DVSSJ2 peptide antibodies were generated as previously reported [1]. For total protein antibody production, the open reading frame of *dvssj1* was inserted into a pDONR221 plasmid and then cloned into C-term BaculoDiret to generate baculoviruses following manufacturer instruction (ThermoFisher). Baculoviruses infected SF9 insect cells and incubated for 72 hours at 27^o^C. Two liters of cell culture pellets were suspended with 200 ml of lysis buffer (1xPBS), then homogenizer twice. Added Urea and CHAPS into lysate to final concentrations of 4 M Urea and 0.5% CHAPS, respectively, and incubated at 4 ^o^C with stirring. The lysate was then centrifuged at 16000 rpm for 20 min. The supernatant (S1) was loaded onto two 2 ml Ni-NTA matrix pre-equilibrated with solubilization buffer (1xPBS, 4 M Urea, 0.5% CHAPS). The column was then washed with 20 ml of Elute buffer (1xPBS, 0.3% CHAPS). Columns were then eluted with 10 ml of elute buffer consisting of 10, 20, 50 and 250 mM of imidazole. Best fractions (E50) were dialyzed against 1XPBS buffer and reloading onto 2-ml NiNTA column. The protein was eluted with 5 ml elute buffer (1xPBS, 0.3% CHAPS, 250 mM imidazole). Samples were analyzed by SDS-PAGE and western blots. Purified DVSSJ1 protein was used to generate the IgG-purified antibody and as a standard for western analyses.

**Optimized method for immunohistochemistry (IHC):** Both total protein and peptide antibodies of DVSSJ1 were used for optimization of immunofluorescence staining in the preliminary experiment. Antibody from the total protein produced clear positive detections of SSJ1 protein in the top part of adjacent epithelial cells (S3A Fig) and selected for immunofluorescence microscopy study. Whole larvae and/or reproductive tissue of adult were dissected in 4% paraformaldehyde in PBS and incubated for 30–60 minutes. After fixing and de-paraffinizing sections, slides were treated with a steamer for target retrieval with 200 ml of 1x target retrieval reagent (Advanced Cell Diagnostics, Hayward, CA) for 15 min. WCR specimens were washed with TNB (0.1M TRIS-HCI, pH 7.5; 0.15M NaCl) and blocked with 2% BSA in TNB with 0.3% Triton X-100. Thereafter, they were incubated with primary antibodies in TNB (1:500) at 4°C overnight. Following five washes (1x PBS), they were incubated with secondary antibodies in TNB (goat anti-mouse antibody Alexa Fluor 488; 1:500) for overnight. After five additional washes (1x PBS), they were coverslipped and imaged with an EVOS FL Auto Imaging System (ThermoFisher) or a confocal laser scanning microscope (TCS SP2; Leica) at room temperature.

**RNA binding to midgut cells:** Fifty diet-raised 2^nd^ instar WCR were transferred to a petri dish containing filter paper circle wetted with sterile 1x PBS +0.5% gentamicin (Sigma) +0.1% antibiotic-antimycotic solution (100x; Sigma) for 2–3 hours. Larvae were surface sterilized by immersion in successive 2-min washes of 70% ethanol and 0.1% Clorox solution and then twice in sterile 1x PBS. Twenty dissected midguts were rinsed in sterile 1X PBS to eliminate gut contents, washed in insect medium (EX-CELL 420 Serum-Free Medium; Sigma) at 1:3 ratio with PBS, and then incubated in batches by treatment per well for 4 hours at 25°C in 24 well tray with 100 µl of insect medium containing 100 µg/µl gentamicin and 1x antibiotic-antimycotic solution. Next guts were incubated with Cy3-labeled *dvssj1* dsRNA and siRNAs at 10 ng/µl in insect medium for 15 hours at 25°C in dark condition. Unincorporated Cy3 dye was used as a control and Cy3 fluorescence intensity of siRNA was adjusted to match the intensity of Cy3-dsRNA by adding non-labeled same siRNA. Midguts were washed twice with 1X PBS and fixed in 4% Paraformaldehyde for 1 hour at room temp. Then midguts were washed three times with 1X PBST (PBS +0.1% Tween) for 5 minutes each. Samples were counterstain with DAPI (Sigma, 10 µg/µl of stock diluted to 1:1000) for 5 min and washed once in 1X PBST. The samples were then coverslipped and imaged on a Leica TCS SPE. The 405 nm laser line was used for the DAPI staining and 532 nm laser line was used with to excite the Cy3 dye. For determination of the fluorescent intensity of the samples, ImageJ (NIH) was used. The 532 nm laser line image was imported and converted to grey scale. The midgut was then selected to exclude other tissue in the image and the mean grey value was calculated for each image. This is done by the sum of the grey values of all the pixels in the selected area is divided by the number of pixels. The mean grey value was compared for each treatment using JMP.

## ***Drosophila melanogaster ssk* knockout and knockin line *via* CRISPER/Cas 9.**

**Strategy and experimental step:** CRISPR/Cas9-mediated genome editing by homology-dependent repair (HDR) using two guide RNAs and a dsDNA plasmid donor was used to replacing *ssk* by homolog *dvssj1* or making ssk deletion mutant at the same breakpoint. The ScarlessDsRed system [2, 3] was employed to facilitate genetic screening using existing gRNAs. *D. melanogaster* strain *w^1118^ was used* for injecting Knock-in cassettes (*dvssj1*-PBacDsRed or PBacDsRed), where the coding region of *ssk* will be replaced by *dvssj1* cDNA or deleted and replaced by selection marker PBacDsRed (S9 Fig). Only one exogenous TTAA motif will be left after *dvssj1* transcript (S10 and S11 Fig). 3XP3-DsRed facilitates the genetic screening and can be excised by Piggy Bac transposase. Genomic DNA was obtained from genomic DNA of injection strain *w^1118^* or final edited lines. PCR was performed using Phusion High-Fidelity DNA Polymerase (Thermo Scientific) on BioRad S1000 Thermal Cycler. Sequencing results were Blat[4]against *D. melanogaster* genome (Aug 2014 Assembly, BDGP Release 6) using UCSC Genome Bioinformatics [5].

**Donor plasmid cloning:** The homology arms of *ssk/CG6981* were amplified by Phusion High-Fidelity DNA Polymerase (Thermo Scientific) from genomic DNA at the optimized condition, which reflects sequences of the injection strain *in vivo*. The upstream and downstream homology arms were submitted for gene synthesis. Cassette PBacDsRed was digested from sequence verified plasmid stock by restriction enzymes. The cassette and two homology arms were cloned by sequence-and ligation-independent method into vector pUC57-Kan, followed by standard transformation protocol, colony PCR selection, and sequencing. Two homology arms and junctions of cassette fragment(s) were confirmed by sequencing. No SNPs are found on upstream and downstream guide RNA in 2 or 3 independent reads in the injection strain. The target sequence of proposed guide RNA is present in the genome of injection strain (not shown). PCR products of balanced lines (heterozygous; TM6B) were shown in S10 Fig. Sequences of edited lines and alignment with donor sequence were shown in S11 Fig.

**Reference:**

1. Hu X, Richtman NM, Zhao JZ, Duncan KE, Niu X, Procyk LA, et al. Discovery of midgut genes for the RNA interference control of corn rootworm. Scientific reports. 2016;6:30542. Epub 2016/07/29. doi: 10.1038/srep30542. PubMed PMID: 27464714; PubMed Central PMCID: PMC4964579.

2. Bier E, Harrison MM, O’Connor-Giles KM, Wildonger J. Advances in Engineering the Fly Genome with the CRISPR-Cas System. Genetics. 2018;208(1):1-18. doi: 10.1534/genetics.117.1113.

3. Gratz SJ, Ukken FP, Rubinstein CD, Thiede G, Donohue LK, Cummings AM, et al. Highly Specific and Efficient CRISPR/Cas9-Catalyzed Homology-Directed Repair in Drosophila. Genetics. 2014;196(4):961-71. doi: 10.1534/genetics.113.160713. PubMed PMID: PMC3982687.

4. Kent WJ. BLAT--the BLAST-like alignment tool. Genome research. 2002;12(4):656-64. Epub 2002/04/05. doi: 10.1101/gr.229202. PubMed PMID: 11932250; PubMed Central PMCID: PMCPMC187518.

5. Kent WJ, Sugnet CW, Furey TS, Roskin KM, Pringle TH, Zahler AM, et al. The human genome browser at UCSC. Genome research. 2002;12(6):996-1006. Epub 2002/06/05. doi: 10.1101/gr.229102. PubMed PMID: 12045153; PubMed Central PMCID: PMCPMC186604.

6. Thompson JD, Higgins DG, Gibson TJ. CLUSTAL W: improving the sensitivity of progressive multiple sequence alignment through sequence weighting, position-specific gap penalties and weight matrix choice. Nucleic Acids Res. 1994;22(22):4673-80. Epub 1994/11/11. PubMed PMID: 7984417; PubMed Central PMCID: PMCPmc308517.
