# Supplementary material for: Molecular characterization of the insecticidal activity of double-stranded RNA targeting the smooth septate junction of western corn rootworm (Diabrotica virgifera virgifera)
Source: PLoS One. 2019 Jan 10;14(1):e0210491. doi: 10.1371/journal.pone.0210491 (PMC6328145; doi:10.1371/journal.pone.0210491)
Supplement: S1 Table — (DOCX) [file pone.0210491.s015.docx]

**S1 Table. Primers, oligos and antibody information**

^a^ TaqMan assay was used for *dvssj1* transcript analyses at a different life stage of WCR on diet; ^b^Triplex TaqMan assay was used for analyses *dvssj1* suppression after dsRNA treatments; ^c^RNA probes were designed by Advanced Cell Diagnostics (Hayward, CA)
