# Supplementary material for: Molecular characterization of the insecticidal activity of double-stranded RNA targeting the smooth septate junction of western corn rootworm (Diabrotica virgifera virgifera)
Source: PLoS One. 2019 Jan 10;14(1):e0210491. doi: 10.1371/journal.pone.0210491 (PMC6328145; doi:10.1371/journal.pone.0210491)
Supplement: S2 Table — (DOCX) [file pone.0210491.s016.docx]

**S2 Table. CRISPER-CAS9 target sites, primer (oligo), and guide RNAs**
